# Reflections on Postpartum Hysterectomy as a Possible Complication of Cesarean Myomectomy: A Long Debate

**DOI:** 10.3390/medicina60040594

**Published:** 2024-04-04

**Authors:** Nikoleta Tabakova, Radmila Sparić, Andrea Tinelli

**Affiliations:** 1 Department of Obstetrics and Gynecology, Medical University Varna, Marin Drinov Street No. 55, 9002 Varna, Bulgaria; 2 Obstetrics and Gynecology Hospital SBAGAL Varna, 9000 Varna, Bulgaria; 3 Faculty of Medicine, University of Belgrade, 11000 Belgrade, Serbia; radmila@rcub.bg.ac.rs; 4 Clinic for Gynecology and Obstetrics, University Clinical Centre of Serbia, 11000 Belgrade, Serbia; 5 Department of Obstetrics and Gynecology, 73100 Scorrano, Lecce, Italy; andreatinelli@gmail.com; 6 CERICSAL (CEntro di RIcerca Clinico SALentino), “Veris delli Ponti Hospital”, 73100 Scorrano, Lecce, Italy

**Keywords:** cesarean myomectomy, postpartum hysterectomy, cesarean section, pregnancy, labor, complications

## Abstract

Uterine fibroids are common benign tumors found in fertile women. Numerous obstetrical issues, such as dystocia during labor, fetal hypotrophy, a ruptured amniotic sac, early labor, low-birth-weight newborns, etc., are associated with fibrous pregnant uteri. Cesarean myomectomy is not a common procedure because of the possibility of postpartum hysterectomy or a potentially lethal hemorrhage. For the chosen topic, we present two instances of emergency postpartum hysterectomies following cesarean myomectomy. After a cesarean myomectomy, two women experienced a perioperative hemorrhage that required a postpartum hysterectomy without a salpingo-oophorectomy. A postpartum hysterectomy was required in every instance due to the failure of additional hemostatic techniques to control the bleeding after the cesarean myomectomy. In every case, the location and number of fibroids—rather than their size—were the primary factors leading to the postpartum hysterectomy. In order to ensure that the patient is safe and that the advantages outweigh the dangers, the current trends in cesarean myomectomy include aiming to conduct the procedure either electively or when it offers an opportunity. The treatment is still up for debate because it is unknown how dangerous a second hysterectomy is for people who have had a cesarean myomectomy.

## 1. Introduction

Uterine fibroids (UFs), or leiomyomas, are the most common benign tumors of female reproductive organs [1]. They are found in smooth muscle cells and connective tissue, and they are steroid-hormone-dependent [1,2]. UFs present mainly in women of reproductive ages. In the past few decades, they have become more and more frequent in pregnant women due to a more advanced age at the time of a woman’s first pregnancy, the progress and wide use of assisted reproductive techniques, and the availability of more advanced diagnostic tools [3]. Hence, the number of pregnant women with fibroids has significantly increased, which adversely affects pregnancy and childbirth. As a result, complications linked to fibroids during pregnancy have also increased, and out of those, red degeneration is the most common [4]. A myomectomy during pregnancy is rarely indicated, except in cases of rapid myoma growth, pain that is resistant to conservative treatment, or an acute abdomen [4]. Even for a skilled obstetrician, treating symptomatic pregnant patients, choosing the best delivery method, and monitoring pregnant women with fibroids can occasionally be quite difficult [5,6,7,8,9,10,11]. Cesarean myomectomy (CM) was introduced into clinical practice over a century ago, but its justifiability is still a matter of debate [12]. A CM is not routinely performed because of the possible risk of life-threatening perioperative complications, such as a hemorrhage or postpartum hysterectomy [13,14,15]. Unfortunately, there is a lack of data from randomized controlled trials concerning the rates of complications during and after CM, even if, for the most part, these are retrospective studies, case collections, or case reports. The published data under-report the number of postpartum hysterectomies performed after CM. Hence, we reviewed the literature reports and analyzed two cases of postpartum hysterectomies following CM as models for evaluating the clinically difficult dilemma.

### 1.1. Case 1

A 32-year-old multigravida patient with two previous deliveries was admitted to a hospital at 37 weeks of gestation due to irregular uterine contractions. The pregnant patient had given birth via cesarean section (CS) in her two previous pregnancies. The patient’s history showed the presence of multiple intramurals and subserous fibroids, diagnosed using an ultrasound in the first trimester. Upon admission, the ultrasound examination showed a transverse lie of the fetus, an intramural fibroid measuring 65 × 50 mm located on the right anterolateral uterine wall, and two other intramural fibroids measuring 50 × 40 mm and 30 × 35 mm, located anteriorly in the lower uterine segment (LUS). The fourth myoma was subserous and located in the uterine fundus, measuring 50 × 40 mm in diameter. When the patient was in labor, the decision was made to perform an urgent CS. A CM was discussed with the patient, and detailed information about its possible complications, including a cesarean hysterectomy, was provided. Informed consent was obtained for both the CS and CM, with an eventual hysterectomy if necessary. The patient’s preoperative hemoglobin level was 10.4 g/dL, and her blood pressure was 110/70 mmHg. In a laparotomy, two fibroids occupying the LUS were recorded; the LUS appeared extremely thin, and the membranes were intact. In order to perform the hysterotomy, it was necessary to perform a myomectomy, and one of the fibroids was removed. A live 3000 g baby was delivered through a transverse LUS incision, which was a separate incision from the one used for the myoma enucleation.

**Figure 1:**
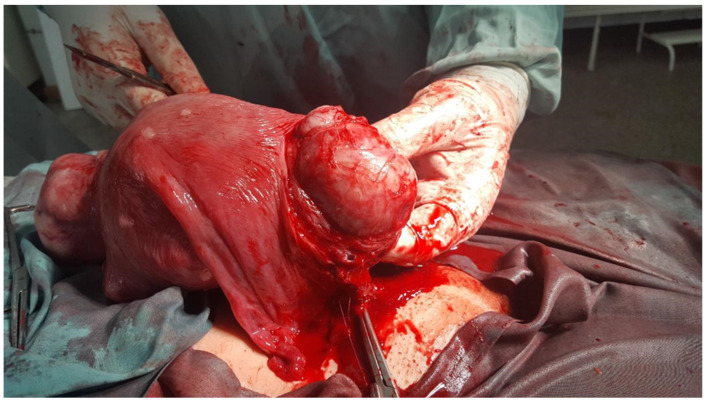
The myomatous uterus right after the delivery of the baby via a cesarean section and during the myomectomy of the fibroids.

**Figure 2:**
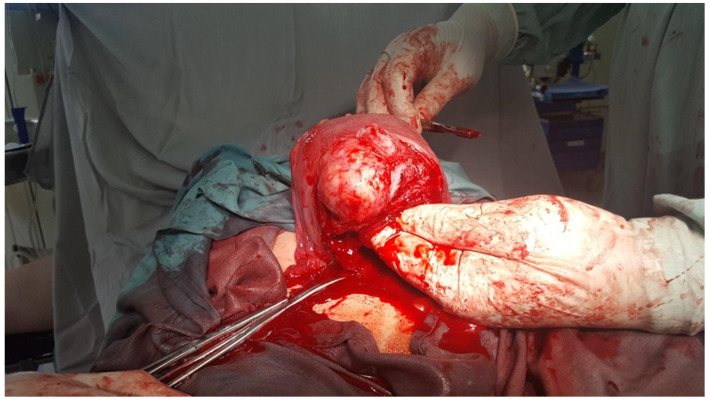
An intramural fibroid located on the level of the lower uterine segment during the cesarean myomectomy.

The hysterorrhaphy was performed in two layers, and successive multiple myomectomies were performed, with the removal of other fibroids: one intramural on the right anterolateral wall, one intramural located on the level of the LUS, and one subserous on the fundus. Additionally, the surgeons detected multiple small fibroids all over the uterus, with an average size of approximately 10 to 20 mm (Figure 1 and Figure 2). After suturing all the fibroid beds, a severe hemorrhage was registered due to an atonic uterus. Since 40 IU of oxytocin was unable to stop the bleeding, intramuscular injections of 1 mg of carboprost trometamol were administered. Additionally, because the estimated blood loss was over 2000 mL, both uterine arteries were clamped; however, this did not stop the bleeding. At 75/50 mmHg, the patient’s blood pressure became hemodynamically unstable, and her hemoglobin level fell to 6.1 g/dL. Therefore, a cesarean hysterectomy was performed without a salpingo-oophorectomy. During the operation, the patient received two units of packed red blood cells (RBCs) and two units of fresh frozen plasma. The entire operation lasted for 190 min. The patient was moved to the hospital’s intensive care unit (ICU), where she received an additional treatment for hypovolemia with another unit of packed red blood cells, fresh frozen plasma, a 50 mL/kg plasma expander solution (HES), 250 mL of thrombocyte concentrate, and a continuous infusion of uterotonics and antibiotics (4 g of ceftriaxone daily), to prevent infections, for three days. The patient was transferred to the maternity unit and discharged home on the eighth postoperative day.

### 1.2. Case 2

**Figure 3:**
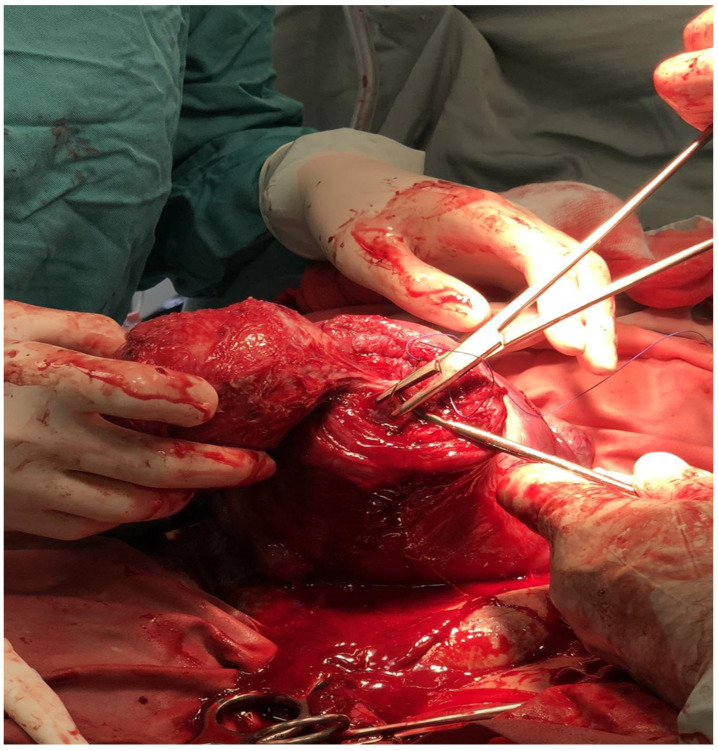
Cesarean myomectomy of intramural fibroid measuring 10 × 9.7 mm.

**Figure 4:**
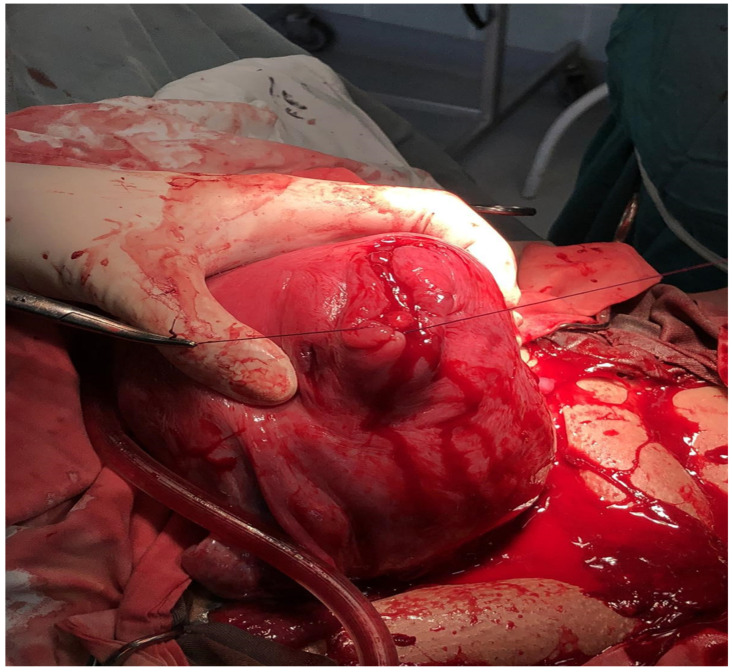
Closure of uterine wall after cesarean myomectomy.

A 37-year-old, gravida 1, para 0 patient was admitted to a university-affiliated hospital for a planned CS at 38 weeks of gestation, for a fetus in an oblique lie and a large myoma. During the antenatal visits, a large intramural fibroid of 100 × 97 mm occupying the anterior uterine wall, extending to the fundus, was diagnosed through an ultrasound. The patient was admitted twice to the hospital in her second trimester, complaining of pain in her lower back and abdomen (presumably caused by the fibroid size). The ultrasound features suggested a fibroid with red degeneration. The patient received analgesics (paracetamol) for the pain. The patient’s preoperative hemoglobin level was 11.0 g/dL. Detailed informed written consent was obtained from the patient for the combined procedure (cesarean section and CM). The patient was given information about the possible complications of the surgery, including a postpartum hysterectomy. A 2570 g live baby was delivered through an LUS incision, which was sutured afterwards in two layers. The myoma was extracted through another incision, which was also sutured in two layers with 1-0 Vicryl (Figure 3 and Figure 4). The uterus contracted under a 20 IU oxytocin infusion and 0.25 mg of carboprost trometamol intramuscularly after the cesarean myomectomy, and the surgery concluded without complications. Six hours later, the patient had profuse vaginal bleeding without signs of uterine atony and developed signs of hemorrhagic shock. Her hemoglobin level was 6.5 g/dL, and her blood pressure was 70/35 mmHg. An emergency relaparotomy was performed, which revealed profuse bleeding from the incision sites. The estimated blood loss was calculated to be an average of over 1500 mL. Due to the inability to perform a hypogastric artery ligation, an immediate postpartum hysterectomy without a salpingo-oophorectomy was successfully performed. During the surgery, the resuscitation measures included an intravenous infusion of two units of RBCs, two units of fresh frozen plasma, 30 UI of oxytocin, and 0.25 mg of carboprost trometamol intramuscularly. The duration of the relaparotomy with the hysterectomy was 130 min. The active monitoring of the patient in the ICU continued with the treatment of the hypovolemia using HES, the prevention of disseminated intravascular coagulation (DIC) using a fresh frozen plasma infusion, and prophylactic antibiotics (2 g of Medaxone daily) for 4 days. Afterwards, the patient was transferred to the maternity unit, where the prophylactic antibiotic treatment continued. The patient was discharged on the seventh postoperative day. The follow-up period was uneventful.

## 2. Discussion

By examining hospital data from 2015 to 2022, the authors found that only two patients out of forty cases—or 5% of all CMs—received postpartum hysterectomy procedures during that time. Therefore, the estimated incidence of postpartum hysterectomies is 2.3/1000 births. The CS rate was 55% over the study period, with 9565 out of 17.391 births resulting in CS. The two postpartum hysterectomies, one in a 32-year-old patient with multiple fibroids and previous CS and the other in a 37-year-old patient with a 100 mm intramural fibroid, were urgently necessary due to the failure of additional hemostatic measures to control the bleeding (Figure 4). To delve into the merits of these two cases, we analyzed the current literature on the complications of CM and subsequent hysterectomies. Only a few cases have been reported in the literature on postpartum hysterectomies as a potential complication of CM.

Sparic et al. described a cesarean hysterectomy case in a 36-year-old after the surgical removal of a large myoma and a subsequent massive hemorrhage from the myoma bed, with hematoma formation in the left retroperitoneal space [16]. 

Li et al. analyzed 1245 CMs and subdivided them into three groups: a CM group, a CS-only group (leaving the myomas in situ), and a cesarean hysterectomy group of women with fibroids. The article did not specify the indications for a cesarean hysterectomy in the group of women with myomas; therefore, we were unable to determine whether or not those hysterectomies were a result of related CMs. Multiple-site fibroids affected the majority of the women who received a cesarean hysterectomy surgery. There were no variations in the frequency of bleeding among the three research groups, according to the authors [17].

Exacoustos and Rosati [18] reported three severe hemorrhages that required life-saving hysterectomies in nine cases of CM. The authors came to the conclusion that, after surgical hemostasis techniques fail, the choice to conduct a hysterectomy during CS should be made carefully. In one of the cases in our study, the surgical occlusion of the uterine arteries failed, and we decided to perform a hysterectomy. 

Out of 23 CM cases, Pattanaik et al. also documented one case of a subtotal hysterectomy following the CM [19]. 

Another study investigated the relaparotomy rate after CS and reported 44 relaparotomies due to a hemorrhage after CM [20].

Tian et al. reported nine cases of CM for cervical fibroids; in one of them, a life-saving postpartum hysterectomy was required due to a massive hemorrhage [21].

The true incidence of postpartum hysterectomies as a complication of CM still remains unknown, probably as a consequence of under-reporting among researchers. On the contrary, some studies have reported a high rate of hysterectomies and postoperative sepsis after CS in conservative cases (without fibroid removal). Large fibroids may increase the likelihood of an emergency hysterectomy by compromising the blood supply to the nearby myometrium and disrupting the oxytocin distribution there, which may result in intra- or peripartum uterine atony [8,22]. After delivery, the uterus becomes less contractile the more fibroids there are, increasing the likelihood of uterine atony and hemorrhage [23].

Tinelli et al. reported a case of a postpartum hysterectomy in a patient with a large myoma left in situ during CS. The authors suggested that the large-sized fibroid was the probable cause of the conservatively uncontrolled postpartum hemorrhage (PPH) and the need for a life-saving hysterectomy. They suggest that further studies on the topic will provide some additional insights regarding the risks of avoiding a CM [24].

### 2.1. The Most Common Complications of a Cesarean Myomectomy

The scientific literature contains studies about complications that follow CM, primarily in the form of retrospective analyses, case reports, and case series, despite the mounting evidence that CM is a safe and feasible operation. Most gynecologists steer clear of CM because of the potential for severe intra- or postoperative bleeding, uterine atony, and a subsequent hysterectomy. Uterine atony is the inability of the uterus to contract immediately after birth and is the most common cause of PPHs, with a reported incidence of approximately 0.05% of all births [25]. When uterine atony is promptly diagnosed, a sequence of steps is started with the goal of compressing the uterus and decreasing the bleeding. Administering high doses of uterotonics (oxytocin, ergometrine, and prostaglandins) is usually the first step. Uterotonics were used as the initial treatment in each of our cases to compress the uterus and halt the bleeding. According to recent studies, there are no appreciable differences in the requirement for extra intraoperative hemostasis procedures between patients who have undergone a CM and those who have only had CS [26].

Akbas et al. investigated the risk of severe intraoperative bleeding and a hysterectomy. The scientists came to the conclusion that the danger increases when large fibroids are removed and when more incisions are required for the removal [27]. Another author concluded that CM should be avoided except for small and pedunculated fibroids [28]. 

Given the numerous cases that have been reported in the scientific literature that support CM, these prior theories have been disproven. According to these data, surgeons have effectively combined hemostatic techniques to lower the possibility of significant intra- or postoperative bleeding. Additionally, the notion that the uterus is adequately supplied with blood throughout pregnancy was prevalent, and as a result, the theoretically high risk of severe intra- and postoperative bleeding led to the rejection of CM [29,30]. For these reasons, most gynecologists have been taught to avoid myomectomy during CS.

Dogan et al. conducted retrospective research on 267 cases of CM and found that, although the patients had low postoperative hemoglobin levels and an increased requirement for blood transfusions, there was no linked risk of hysterectomies or other life-threatening complications [31]. After a CM, when low hemoglobin levels and high blood loss occur together, it is natural to expect anemia and the need for blood transfusions. 

In recent years, an increased incidence of uterine atony after myomectomies has been reported. A meta-analysis on the post-CM uterine atony rate indicated a percentage of 0.6–0.75% [32,33]. Hsieh et al. analyzed 47 incidental myomectomies performed during CSs, reporting that such a combined intervention increases the average blood loss after the CS by 112 mL, prolongs the operative time by 11 min, and increases the hospital stay by half a day [14].

A case series on 10 cases of CM with single anterior-wall fibroids indicated only two cases of PPH, one of which involved severe blood loss of over 2000 mL, requiring a blood transfusion and a uterine tourniquet placement as a hemostatic measure [34].

The study by Sparic et al. on intraoperative blood loss as a risk factor for complications during CM showed that blood loss above 500 mL was of primary importance [35].

A Chinese study reported, as an unfavorable outcome of CM, a high risk for PPH involving blood loss of >1000 mL associated with large fibroids (>50 mm) [36].

Similar results were shared by Askin et al. in their study on the assessment of intra-and postoperative results of CM; they found no statistical differences between patients undergoing CM or only CS in terms of hemoglobin changes, the need for a blood transfusion, and the operation time [37].

Tabakova et al. detected that the mean intraoperative blood loss between patients receiving CS alone and those receiving CM did not differ significantly [38]. Ramesh et al. retrospectively analyzed 21 CM cases and did not find an association with intraoperative and postoperative complications [39]. A systematic review did not find an incidence of any major perioperative complications of CM in comparison with CS alone [40].

A recent study analyzing CM cases published in the last 10 years did not find any clinically significant differences in the intraoperative blood loss, hospital stay length, or operative times between two groups—4702 (71.85%) women who received CM and 1843 (28.15%) women who received CS only [41]. On the contrary, a meta-analysis that included a total of 8521 patients, of whom 5586 underwent CM and 2935 underwent CS only, reported statistically significant differences in terms of the hemoglobin changes, intraoperative blood loss, transfusion rates, and duration of the hospital stay [42].

Similar results were reported by Huang et al. in a meta-analysis of 23 studies on CM, with a significant drop in hemoglobin values, a longer hospital stay, and a high incidence of PPH in the CM group vs. the CS-only group [43].

A recent study comparing the features of fibroids and postoperative complications found that only large (>100 mm) and heavy (>500 g) fibroids were associated with an increased predicted blood loss and post-CM blood transfusion risk [44]. On the contrary, our results show a link between postpartum hysterectomies and the number and location of the fibroids rather than their size.

### 2.2. Treatment Options for Bleeding Following CM That Preserve the Uterus

In respect to intra- and postoperative blood loss during CM, Sapmaz et al. examined the impact of a bilateral tourniquet on the ascending branch of the uterine artery following CS and before CM, compared to just its bilateral closure. The intraoperative blood loss outcomes for both techniques did not show a substantial difference. To prevent the rupturing of the broad ligament and big vessels, as well as the formation of postoperative adhesions, both procedures call for dexterity and caution [45].

Some gynecologists believe that methods for temporarily blocking the uterine arteries, such as clamping or local vasopressin injections into the myometrium, are needless and even detrimental to the healthy healing of the myometrium. The masking of later bleeding from the momentarily collapsed arteries is another drawback of these procedures. 

According to Tinelli et al. [46], a modern myomectomy should be performed without compromising the blood flow to the uterus (“beating heart” surgery), thus avoiding other disadvantages of hemostatic operative techniques, such as (a) bleeding from the cicatrix due to revascularization, (b) a possible increased incidence of postoperative adhesions, (c) a negative impact on successive fertility (as there is a lack of evidence that uterine artery occlusion has such an impact), and (d) the cardiovascular side effects of vasopressin. The authors are of the opinion that all methods for the temporary occlusion of uterine blood flow should be used only for the enucleation of deep intramural fibroids [46].

One of the main disadvantages of CM comes from the fact that, until now, reliable data from large randomized multicenter trials have been missing. Furthermore, our opinion is that the rate of hysterectomies associated with CM is under-reported in case reports. 

This large debate should continue in scientific societies concerning the recommendations for the use of CM during delivery as a standard operative procedure with the prior selection of patients. 

## 3. Conclusions

The number of pregnant people with fibroids, which adversely affect the obstetric outcome, has significantly increased, as has the age of women at the time of their first pregnancy. Pregnancies complicated by fibroids are at a higher risk, not only because of the potential complications due to the uterine mass, but also because of other associated co-factors, such as an advanced maternal age, obesity, in vitro fertilization, etc. The prevalence of lower-segment cesarean sections has also increased worldwide. Based on these facts, the need to perform myomectomies during CS should also increase. In order to consider performing a CM while reducing the risk of complications, in the absence of effective guidelines, some features must be evaluated. A universal panel for determining whether or not to recommend a procedure does not yet exist, but it is possible to follow some generic recommendations, such as having well-trained obstetric surgeons in the operating room who thoroughly know the techniques and complications of a CM, correctly selecting patients, and using a wide range of hemostatic techniques on the myometrium. Nevertheless, despite these safety principles, myomectomy during CS remains a controversial issue in obstetric practice and will continue to be debated in the future.

## Data Availability

Not applicable.

## List of Abbreviations

Uterine fibroids	UFs
Cesarean myomectomy	CM
Cesarean section	CS
Lower uterine segment	LUS
Red blood cells	RBCs
Intensive care unit	ICU
Plasma expander solution	HES
Disseminated intravascular coagulation	DIC
Postpartum hemorrhage	PPH

## References

[1] Sparic R., Mirkovic L., Malvasi A., Tinelli A. (2016). Epidemiology of Uterine Myomas: A Review. Int. J. Fertil. Steril..

[2] Tinelli A., Sparic R., Kadija S., Babovic I., Tinelli R., Mynbaev O.A., Malvasi A. (2016). Myomas: Anatomy and related issues. Minerva Ginecol..

[3] Sparić R., Malvasi A., Tinelli A. (2015). Analysis of clinical, biological and obstetric factors influencing the decision to perform cesarean myomectomy. Ginekol. Pol..

[4] Sparić R. (2014). Uterine myomas in pregnancy, childbirth and puerperium. Srp. Arh. Celok. Lek..

[5] Klatsky P.C., Tran N.D., Caughey A.B., Fujimoto V.Y. (2008). Fibroids and reproductive outcomes: A systematic literature review from conception to delivery. Am. J. Obstet. Gynecol..

[6] Chuang J., Tsai H.W., Hwang J.L. (2001). Fetal compression syndrome caused by myoma in pregnancy, a case report. Acta Obstet. Gynecol. Scan..

[7] Aydeniz B., Wallwiener D., Kocer C., Grischke E.M., Diel I.J., Sohn C., Bastert G. (1998). Der Stellenwert myombedingt Komplikationen in der Schwangerschaft. Z. Geburtshilfe Neonatol..

[8] Hasan F., Arumugam K., Sivanesaratnam V. (1991). Uterine leiomyomata in pregnancy. Int. J. Gynecol. Obstet..

[9] Winer-Muram H.T., Muram D., Gillieson M.S. (1984). Uterine myomas in pregnancy. J. Can. Assoc. Radiol..

[10] Vergani P., Ghidini A., Strobelt N., Roncaglia N., Locatelli A., Lapinski R.H., Mangioni C. (1994). Do uterine leiomyomas influence pregnancy outcome?. Am. J. Perinatol..

[11] Girault A., Le Ray C., Chapron C., Goffinet F., Marcellin L. (2018). Leiomyomatous uterus and preterm birth: An exposed/unexposed monocentric cohort study. Am. J. Obstet. Gynecol..

[12] Sparić R., Malvasi A., Kadija S., Babović I., Nejković L., Tinelli A. (2017). Cesarean myomectomy trends and controversies: An appraisal. J. Matern.-Fetal Neonatal Med..

[13] Burton C.A., Grimes D.A., March C.M. (1989). Surgical management of leiomyomata during pregnancy. Obstet. Gynecol..

[14] Hsieh T.T., Cheng B.J., Liou J.D., Chiu T.H. (1989). Incidental myomectomy in cesarean section. Changgeng Yi Xue Za Zhi.

[15] Michalas S.P., Oreopoulou F.V., Papageorgiou J.S. (1995). Myomectomy during pregnancy and caesarean section. Hum. Reprod..

[16] Sparić R., Radunović N., Tinelli A., Radević O., Kadija S. (2018). Large myomas as a complicating factor necessitating cesarean myomectomy followed by cesarean hysterectomy. Srp. Arh. Celok. Lek..

[17] Li H., Du J., Jin L., Shi Z., Liu M. (2009). Myomectomy during cesarean section. Acta Obstet. Gynecol. Scand..

[18] Exacoustòs C., Rosati P. (1993). Ultrasound diagnosis of uterine myomas and complications in pregnancy. Obstet. Gynecol..

[19] Pattanaik T., Kumar Pati B., Samal S. (2014). Caesarean myomectomy: A descriptive study of clinical outcome. Int. J. Reprod. Contracept. Obstet. Gynecol..

[20] Seffah J.D. (2005). Re-laparotomy after Cesarean section. Int. J. Gynaecol. Obstet..

[21] Tian J., Hu W. (2012). Cervical leiomyomas in pregnancy: Report of 17 cases. Aust. N. Z. J. Obstet. Gynaecol..

[22] Dedes I., Schaffer L., Zimmermann R., Burkhardt T., Haslinger C. (2017). Outcome and risk factors of cesarean delivery with and without cesarean myomectomy in women with uterine myomatas. Arch. Gynecol. Obstet..

[23] Koike T., Minakami H., Kosuge S., Usui R., Matsubara S., Izumi A., Sato I. (1999). Uterine leiomyomas in pregnancy: Its influence on obstetric performance. J. Obstet. Gynecol..

[24] Tinelli A., Nezhat C., Likić-Ladjević I., Andjić M., Tomašević D., Papoutsis D., Stefanović R., Sparić R. (2021). Myomectomy during cesarean section or non-caesarean myomectomy in reproductive surgery: This is the dilemma. Clin. Exp. Obstet. Gynecol..

[25] Holmgren C.M. (2012). Uterine rupture associated with VBAC. Clin. Obstet. Gynecol..

[26] Dai Q.H., Zhang L., Chen A.E. (2024). Prognostic and reproductive outcomes in women who had uterine myomas removed during cesarean section and sutured using different techniques. BMC Women’s Health.

[27] Akbas M., Mihmanli V., Bulut B., Temel Yuksel I., Karahisar G., Demirayak G. (2017). Myomectomy for intramural fibroids during caesarean section: A therapeutic dilemma. J. Obstet. Gynaecol..

[28] Ortac F., Güngőr M., Sőnmezer M. (1999). Myomectomy during cesarean section. Int. J. Gynecol. Obstet..

[29] Okogbo F.O., Ezechi O.C., Loto O.M., Ezeobi P.M. (2011). Uterine leiomyomata in South Western Nigeria: A clinical study of presentations and management outcome. Afr. Health Sci..

[30] Velasco V.R. (2006). Update of the conservative management of the uterine leiomyomata. An. Real Acad. Nac. Med..

[31] Doğan S., Özyüncü Ö., Atak Z. (2016). Fibroids during pregnancy: Effects on pregnancy and neonatal outcomes. J. Reprod. Med..

[32] Claeys J., Hellendoorn I., Hamerlynck T., Bosteels J., Weyers S. (2014). The risk of uterine rupture after myomectomy: A systemic review of the literature and meta-analysis. Gynecol. Surg..

[33] Kim H.S., Oh S.Y., Choi S.J., Park H.S., Cho G.J., Chung J.H. (2016). Uterine rupture in pregnancies following myomectomy: A multicenter case series. Obstet. Gynecol. Sci..

[34] Amatya Vaidya S., Jha R. (2021). Caesarean Myomectomy among Patients Undergoing Lower Segment Caesarean Section in a Tertiary Care Center. J. Nepal Med. Assoc..

[35] Spariç R. (2016). Intraoperative hemorrhage as a complication of cesarean myomectomy: Analysis of risk factors. Vojnosanit. Pregl..

[36] Zhao R., Wang X., Zou L., Zhang W. (2019). Outcomes of Myomectomy at the Time of Cesarean Section among Pregnant Women with Uterine Fibroids: A Retrospective Cohort Study. BioMed Res. Int..

[37] Guler A.E., Guler Z.Ç.D., Kinci M.F., Mungan M.T. (2020). Myomectomy During Cesarean Section: Why Do We Abstain from?. J. Obstet. Gynecol. India.

[38] Tabakova N. Myomectomy during Abdominal Delivery. https://repository.mu-varna.bg/handle/nls/618.

[39] Ramesh Kumar R., Patıl M., Sa S. (2014). The utility of cesarean myomectomy as a safe procedure. J. Clin. Diagn. Res..

[40] Pergialiotis V., Sinanidis I., Louloudis I.E., Vichos T., Perrea D.N., Doumouchtsis S.K. (2017). Perioperative Complications of Cesarean Delivery Myomectomy: A Meta-analysis. Obstet. Gynecol..

[41] Goyal M., Dawood A.S., Elbohoty S.B., Abbas A.M., Singh P., Melana N., Singh S. (2021). Cesarean myomectomy in the last ten years; A true shift from contraindication to indication: A systematic review and meta-analysis. Eur. J. Obstet. Gynecol. Reprod. Biol..

[42] Youshanloie M., Vaezi M., Pashazadeh F. (2023). Consequences of Concurrent Myomectomy and Caesarean Section versus Caesarean Section Alone in the Last Two Decades: Systematic Review and Meta-Analysis. Curr. Women’s Health Rev..

[43] Huang Y., Ming X., Li Z. (2020). Feasibility and safety of performing cesarean myomectomy: A systematic review and meta-analysis. J. Matern.-Fetal Neonatal Med..

[44] Lee Y.E., Park S., Lee K.Y., Song J.E. (2023). Risk factors based on myoma characteristics for predicting postoperative complications following cesarean myomectomy. PLoS ONE.

[45] Sapmaz E., Celik H., Altungül A. (2003). Bilateral ascending uterine artery ligation vs. Tourniquet use for hemostasis in cesarean myomectomy. A comparison. J. Reprod. Med..

[46] Tinelli A. (2022). Bleeding during laparoscopic myomectomy? It depends on the biology-based technique. Fertil. Steril..

